# The effect of pain on reference memory for duration

**DOI:** 10.1007/s00426-021-01508-3

**Published:** 2021-04-01

**Authors:** Andrea Piovesan, Laura Mirams, Helen Poole, Ruth Ogden

**Affiliations:** School of Psychology, Liverpool John Moores University, Liverpool, UK

## Abstract

Previous research has consistently reported that pain related stimuli are perceived as lasting longer than non-pain related ones, suggesting that pain lengthens subjective time. However, to date, the investigation has been limited to the *immediate* effects of pain on time perception. The current study aims to investigate whether pain affects how a duration is recalled after a period of delay. In two experiments, participants were asked to complete four temporal generalisation tasks, where they were required first to remember the duration of a standard tone (learning phase) and then to compare the standard duration to a series of comparison durations (testing phase). Using a 2 × 2 design, the four tasks differed in terms of whether participants were exposed to a painful or non-painful stimulus during the learning phase, and whether the testing phase started immediately or 15 min after the learning phase. Participants were exposed to low pain in Experiment 1 and high pain in Experiment 2. Two possible results were expected: pain could decrease temporal accuracy, because pain disrupts cognitive processes required for accurate timing, or pain could increase temporal accuracy, because pain facilitates memory consolidation. Contrary to expectations, results from both Experiments indicated that participants’ temporal performances were similar in the pain and no-pain conditions when testing occurred 15 min after the learning phase. Findings, therefore, suggest that pain neither disrupts nor enhances long-term memory representations of duration.

## Introduction

Temporal distortions pervade our daily experience and are well evidenced in laboratory studies. Numerous variables have the capacity to distort the perceived duration of events. For example, duration is subjectively lengthened by visual flickers (e.g., Ortega & López, 2008), click-trains (e.g., Penton-Voak et al., 1996) and negative emotional arousal (e.g., Grommet et al., 2011). In contrast, duration is subjectively shortened by positive stimuli (e.g., Ogden et al., 2015) and shameful facial expressions (e.g., Gil & Droit-volet, 2011). Pain, in particular, has shown the most distorting effects on time perception (e.g., Rey et al., 2017).

Numerous studies show that pain distorts perceptions of time. For example, in a temporal bisection task, which involved categorising a series of comparison durations as more similar to a previously learnt short or long duration, participants gave longer temporal judgments in trials that included the presentation of an electric shock (Fayolle, Gil, & Droit-Volet, 2015). The effect of pain on perceived duration has a magnitude that is typically greater than that observed for negatively valenced visual and auditory stimuli (Gil & Droit-Volet, 2011; Ogden, Moore, et al., 2014; Ogden, Wearden, & Montgomery, 2014). Furthermore, the extent of temporal distortions due to a painful stimulus increases with pain intensity (Piovesan et al., 2019).

The distortions to time evoked by pain experience are often understood within the framework of Scalar Expectancy Theory (SET, Gibbon, Church, & Meck, 1984). SET proposes that time perception and timed behaviour are accomplished by three distinct processes. The raw representation of time is encoded by a pacemaker accumulator clock. According to SET, at the start of a to-be-timed event, output from the pacemaker is transferred to the accumulator via the closure of the switch. The amount of accumulated output forms the representation of duration, with a greater level of accumulation indicating longer duration. This information is then transferred to short term memory (STM), for use in single trials, and reference memory, which is part of the long-term memory (Cassel & Pereira de Vasconcelos, 2015), for use over longer periods of time or multiple trial. The contents of STM and reference memory are then compared by some decision threshold to enable behavioural output.

Within the SET framework, temporal distortions caused by pain are usually explained by a change in the rate at which the internal clock emits output (Fayolle et al., 2015). This change is thought to occur, because pain experience increases physiological arousal, which in turn increases the output rate of the putative clock (see Piovesan et al., 2019; Ogden, Henderson, et al., 2019; Ogden, MacKenzie-Phelan, et al., 2019 for discussion). However, while there is good evidence for pain altering internal clock speed, it is unclear whether pain influences the operation of other component processes of SET, for example the memorization of duration. This is in part, because research has focused on examining the effect of pain on the immediate perception of duration in which memory load is low. No research, to date, has tested how pain may influence the retention of duration information in long-term reference memory over a period of delay.

There are two potential ways in which pain may affect the memorization of duration information. (1) Pain may *disrupt* encoding to, retention in and retrieval from reference memory, leading to an impairment in memory for duration. (2) Pain may *enhance* encoding to, retention in and retrieval from reference memory, improving memory for duration. These possibilities are discussed below.

Pain may be expected to impair memory for duration, because it affects the general cognitive processes upon which temporal processing is reliant (Buhle & Wager, 2010; Eccleston & Crombez, 1999). Accurate temporal processing requires sufficient attention, working memory and executive function (Brown, 2006; Ogden, Moore, et al., 2014; Ogden, Wearden, et al., 2014; Zélanti & Droit-Volet, 2011). When these resources are exceeded or impaired timing is disrupted, becoming more variable and less accurate (Brown, 1997; Ogden et al., 2011), possibly because (1) representations of duration in reference memory are themselves more variable, or because (2) they are more difficult to retrieve from reference memory when working memory and executive resources are limited (Ogden et al., 2014; Ogden, Wearden, et al., 2014).

Indeed, a study showed that nurses who were asked to memorise a 4-s duration and to recall it after 24 h, provided less accurate and more variable responses if they were exposed to high levels of stress during the 24-h delay, perhaps due to reduced attentional resources (Cocenas-Silva, Droit-Volet, & Gherardi-Donato, 2019). It is also well established that pain impairs the maintenance of items in memory (Dick & Rashiq, 2007) and recognition accuracy (Forkmann et al., 2016). Pain also impairs attentional processing (Moore, Keogh, & Eccleston, 2012; Van Damme, Crombez, & Lorenz, 2007) and executive function (Moriarty, McGuire, & Finn, 2011). Leavitt and Katz (2006) suggested that pain affects memory processes, possibly because pain functions as a distractor leading to reduced attentive resources dedicated to the experimental task. It is, therefore, possible that pain may impair the attentional, memory and executive resources required to encode and maintain duration representations in memory, leading to impaired future recall of duration.

Conversely, however, it is possible that pain may enhance the accuracy of memory for duration. In general cognition, memories for emotional events are often superior to those for neutral events (Brown & Kulik, 1977; Kensinger & Corkin, 2003; Lindström & Bohlin, 2011). Temporal memories also appear to be enhanced by emotions. Cocenas-Silva Bueno, and Droit-Volet (2012) used a temporal generalisation task to examine how memory for the perceived duration of emotional and neutral events was affected by a delay between encoding and recall. While perceived duration of neutral stimuli was subjectively longer after a 24-h delay than after immediate encoding, the perceived duration of emotional stimuli was not distorted following the 24-h delay. Furthermore, variability of temporal judgments after the 24-h delay was greater for neutral stimuli than for emotional stimuli. The improvement of emotions on memory for duration was found to also persist after 6 months delay between training and testing (Rattat & Droit-Volet, 2007). Like other forms of memory, memory for duration, therefore, appears to be less vulnerable to distortion and decay when emotional than when neutral.

Emotions may enhance memory for duration, because emotions promote the release of the adrenal stress hormones that facilitate memory consolidation by the hippocampus (LaBar & Cabeza, 2006; McGaugh, 2000). This results in arousing emotions enhancing memory for events (D’Argembeau & Van der Linden, 2004; Dunbar & Lishman, 1984; Sharot & Phelps, 2004). Similar to emotion, pain also is a highly arousing experience and promotes the release of adrenal stress hormones (Bear, Connors, & Paradiso, 2007) and, therefore, may also be expected to enhance memory for duration.

Establishing the way in which pain influences memory for duration is important if we are to develop a complete picture of the way in which emotional somatosensory stimuli distort time. The current study, therefore, sought to establish how durations stored in reference memory were influenced by the experience of pain during the encoding of duration information.

The current study tested the effect of experiencing low pain (Experiment 1) and high pain (Experiment 2) during the encoding of a non-painful temporal stimulus on immediate and delayed temporal generalisation performance. In each experiment, participants completed four temporal generalisation tasks. Each task was split into two phases, a learning phase and a testing phase. In the learning phase, the participants’ task was to memorize the duration of a tone (standard duration), while they experienced either (1) painful stimulation on their arm or (2) neutral stimulation on their arm. In the testing phase, the participants’ task was to indicate whether a series of comparison durations were the same duration as that presented in the learning phase. The testing phase either occurred immediately after the learning phase or following a 15-min delay. A 15-min delay was selected on the basis of previous research indicating that this is sufficient for memory consolidation to take place (Lechner, Squire, & Byrne, 1999) and on the basis of temporal studies demonstrating significant memory deterioration following a 15-min delay (e.g., Lieving et al., 2006; Wearden & Ferrara, 1993; Wearden, Parry, & Stamp, 2002). No additional (i.e., painful or neutral) stimulation was experienced during the testing phase. All participants, therefore, completed four versions of this task (i) no-pain immediate testing, (ii) no-pain delayed testing, (iii) pain immediate testing and (iv) pain delayed testing.

It was expected that the 15-min delay would decrease temporal accuracy and temporal discrimination in the no-pain condition, confirming previous studies (e.g., Lieving et al., 2006; Wearden & Ferrara, 1993; Wearden et al., 2002). Second, two potential outcomes were hypothesised for the effect of pain on immediate and delayed testing. Learning a duration in a state of pain could either *impair* memory processing, leading to poorer recognition of the learnt duration and more variable, less accurate responding in comparison with learning the duration in a neutral state. Alternatively, learning a duration in a state of pain could *enhance* memory processing of the duration, leading to better identification of the learnt duration and less variable, more accurate responding in comparison with learning the duration in a neutral state.

## Experiment 1

### Method

#### Participants

Twenty-eight participants (18 females and 10 males; mean age = 25.79, SD = 6.05) were recruited. The sample size was based on previous studies investigating the effect of pain on perceived duration (Ogden, Moore, et al., 2014; Ogden, Wearden, et al., 2014; Piovesan et al., 2019) and on memory for duration (Cocenas-Silva et al., 2013). A post-hoc power analysis with an alpha of 0.05, a power of 0.95, 1 number of groups, and 28 number of measurements using G*Power (versions 3.1.9.7; Faul et al., 2007), indicated that the study had sufficient participants to detect a small effect size (*f* = 0.15). Participants were required not to be pregnant and not to have chronic pain, skin problems (e.g., eczema) or any impairment of body sensation. Additionally, they were asked not to take any analgesic during the 8 h prior to the experiment. Participants were reimbursed £5 in vouchers for taking part. The study was approved by the Liverpool John Moores University ethics committee and informed consent was obtained from all participants. The study was conducted in accordance with the declaration of Helsinki.

#### Apparatus and materials

A Medoc PATHWAY-Advanced Thermal Stimulator was used to induce pain stimulation. This equipment, used in clinical and research settings, induces pain through a thermode, placed on the skin. Specialist hardware and software, designed for experimental purposes, delivered and controlled the temperature of the thermode. Here, the thermode consisted of a 30 × 30 mm Peltier metal plate attached to the participants’ left volar forearm. This equipment is able to increase the temperature at a ramp rate up to 8 °C/s and to decrease it at a ramp rate of 4 °C/s.

#### Procedure

Participants were initially asked to complete a health screening questionnaire to confirm their suitability to participate. Participants then performed an intensity rating task to establish the thermode intensities to be used in the following four temporal generalisation tasks. Finally, participants were debriefed.

##### Intensity rating task

A search protocol was used to establish the two subjective intensity levels of stimulation (i.e., no pain and low pain) that were used in the following temporal generalisation tasks. After the thermode was applied to the participant’s left volar forearm, participant’s task was to use the 11-point Numeric Rating Scale (NRS, Jensen & McFarland, 1993; 0 = no pain at all, 10 = worst pain imaginable) to identify the thermal intensities equal to 0 (no pain) and 3 (low pain; following Serlin et al., 1995). Participants were instructed to press a mouse button to increase the temperature of the thermode, which started from a baseline temperature of 32 °C and increased approximately 0.10 °C after each time the participant pressed the button. Participants’ aim was to increase the temperature of the thermode until it was considered warm but not painful (as 0 on the NRS). The selected temperature was maintained for 15 s before participants were asked whether the sensation was still at the same intensity. If participants indicated that the sensation changed, they were asked to adjust the temperature and this check was performed again until a reliable percept was reached. Participants were then asked to repeat this procedure to reach an intensity level equal to 3 on the NRS. The thermode never exceeded a temperature of 48 °C to ensure participants’ safety.

##### Temporal generalisation task

After the intensity rating task, participants completed the four temporal generalisation tasks: (i) no-pain immediate testing (ii) pain immediate testing, (iii) no-pain delayed testing and (iv) pain delayed testing. The order of these tasks was counterbalanced across all participants and were administered using E-Prime software (http://www.pstnet.com). The basic task structure consisted of learning and testing phases. In the learning phase, participants were told that they would be presented with a standard tone three times and that their task was to remember how long the tone lasted for. The number of standard presentations (3) was selected to be consistent with previous studies (Ogden, Henderson, et al., 2019; Ogden, MacKenzie-Phelan, et al., 2019; Ogden, Moore, et al., 2014; Ogden, Wearden, et al., 2014), but it should be noted that Ogden and Jones (2009) demonstrated that the number of standard presentations does not affect the temporal performance. The standard was presented as a 500 Hz tone and its duration was randomly selected from a normal distribution from 400 to 800 ms. This ensured that participants were presented with different standard durations across the four tasks, so to avoid learning effect. Each presentation was preceded by an inter-trial interval randomly selected from a 2500–3000 ms range. In the testing phase, participants were informed that they would be presented with a series of comparison tones and that their task was to decide whether each tone was the same length as the standard tone that they previously learnt pressing ‘Y’ for yes or ‘N’ for no. At the start of each trial, participants were instructed to press the spacebar. A comparison stimulus was then presented in the form of a 500 Hz tone and participants indicated whether the comparison tone had the same duration as the standard. Between trials, a delay randomly selected from a 1000–1500 ms range was then interposed. On each trial, the duration of the comparison stimulus was determined by multiplying the standard by 0.625, 0.750, 0.875, 1, 1.125, 1.250 or 1.375. Each of these comparison durations was presented once per block of trials, apart from comparison stimulus duration ‘1′, which was repeated 3 times. This resulted in 9 stimuli per block. A total of six blocks were presented in each task giving a total of 54 stimuli per task. No performance feedback was given to participants.

In the pain conditions, participants felt the thermode being at the low pain intensity during the learning phase. The thermode started with a baseline temperature of 32 °C. At the beginning of the learning phase, the thermode increased its temperature at a ramp rate of 8 °C/s until reaching the low pain intensity selected by participant during the intensity rating task. The standard tone was presented for the first time after 3 s from the initial temperature increase of the thermode, allowing the thermode to reach the target temperature. After 15 s from the initial temperature increase of the thermode, the temperature decreased at a ramp rate of 4 °C/s until reaching again 32 °C. No thermal stimulation was presented during the testing phase.

In the no-pain conditions, the procedure was the same as that used in the pain conditions with the exception of the target temperature; here, the thermode increased its temperature until reaching the no-pain intensity selected by participant in the intensity rating task. No thermal stimulation was presented during the testing phase.

In the delayed testing conditions, a 15-min delay was interposed between the learning and testing phases. During this 15-min delay, participants listened to either “The Wizard of OZ” or “The Jungle Book”. The audiobook assigned to the no-pain delayed testing condition or to the pain delayed testing condition was counterbalanced across participants. This task was chosen rather than a cognitive task to avoid interference on the working memory or executive functions (Mirams et al., 2013).

#### Data analysis

From the temporal generalisation task, we extrapolated the temporal gradients as the proportion of YES responses (i.e., identification of comparisons as the standard) given by each participant in each of the conditions. In addition, measures of response accuracy, response variability, peak time and response spread (indexed by Full Width at Half-Maximum; FWHM) were calculated for each participant in each condition as in Ogden, Henderson, et al. (2019), Ogden, MacKenzie-Phelan, et al. (2019)) and in Hinton and Rao (2004).

##### Accuracy

Accuracy was calculated as the sum of hits and correct rejections divided by 2. Hits corresponded to proportion of YES responses when the comparison’s duration was equal to the standard (i.e., 1). Correct rejections corresponded to proportion of NO responses when the comparison’s duration was not equal to the standard (i.e., 0.625, 0.750, 0.875, 1.125, 1.250 and 1.375). Here a higher score indicates greater accuracy.

##### Variability

The mid-three measure used in Ogden, Henderson, et al. (2019), Ogden, MacKenzie-Phelan, et al. (2019)) and Wearden et al. (1997) was calculated. Mid-three is an index of response dispersion and was calculated as the sum of proportion of YES responses in the three middle comparisons (i.e., 0.875, 1 and 1.125) divided by the sum of YES responses of all comparisons. Higher mid-three scores are taken to indicate that gradients are more peaked around the standard and that participants have greater temporal discrimination.

##### Peak time

The peak time used in Hinton and Rao (2004) was calculated with the Excel Solver Add-in by fitting each participant’s temporal gradient with the Gaussian curve using a least-squares method to minimize the residuals. The peak time indicates the stimulus duration that gave rise to the highest proportion of “same” responses. Higher peak time indicates longer perceived duration of the standard stimulus, and a peak time of 1 indicates that participants were accurate in discriminating the correct comparison (1) as similar to the standard.

##### FWHM

Together with the peak time, the Full Width at Half-Maximum (FWHM) used in Hinton and Rao (2004) was calculated with the Excel Solver Add-in by fitting each participant’s temporal gradient with the Gaussian curve using a least-squares method to minimize the residuals. The FWHM is an indication of the spread of participant’s responses and is the width of a fitted curve measured between those points on the *y*-axis which are half the maximum amplitude. Higher FWHM indicates greater spread of participant’s responses.

Throughout the analyses, Greenhouse-Geisserr correction was applied to ANOVAs when the Sphericity assumption was violated and post-hoc were Bonferroni corrected. Additionally, Bayesian factors (BF) were calculated using JASP (version 0.14.1) when statistical analyses were not significant to indicate how much more likely the data were under the null hypothesis compared to the alternative hypothesis. Lower BF indicates higher likelihood of the null hypothesis (BF < 0.01: extreme; 0.01 < BF < 0.03: very strong; 0.03 < BF < 0.1: strong; 0.1 < BF < 0.33: moderate; 0.33 < BF < 1: anecdotal; Lee & Wagenmakers, 2014).

### Results

On average, participants selected 37.63 °C (SD = 1.91) as no-pain intensity and 42.77 °C (SD = 1.40) as low pain intensity during the initial intensity rating task. A paired-sample t-test indicated that participants selected a significantly higher temperature for the pain conditions compared to the no pain conditions (*t*(27) = 15.62, *p* < 0.001).

**Fig. 1:**
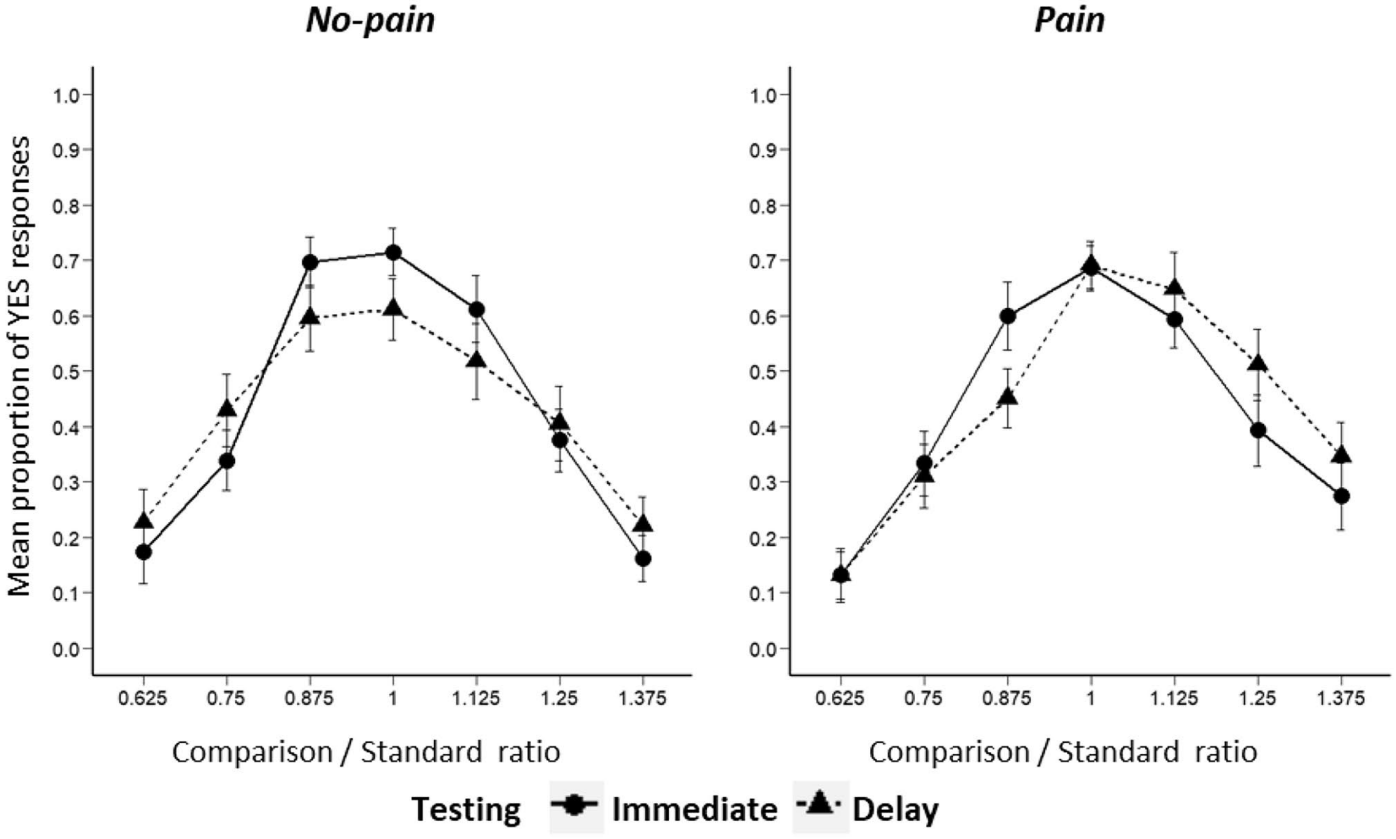
Proportion of YES responses plotted against comparison/standard ratio in Experiment 1. YES responses are divided between immediate (*solid line*) and delay (*dotted line*), and between no-pain (*left panel*) and pain (*right panel*)

Figure 1 shows temporal generalisation gradients depicting the mean proportion of YES responses in the four conditions plotted against comparison/standard ratio. A repeated measures ANOVA with pain intensity (no-pain vs pain), delay (immediate vs delay) and comparison/standard ratio (0.625, 0.750, 0.875, 1, 1.125, 1.250 or 1.375) as within-subject factors was conducted. There was a significant main effect of ratio (*F*(2.02, 54.46) = 32.77, *p* < 0.001, *η*_*p*_^2^ = 0.55) on the proportion of YES responses. There was no significant main effect of pain intensity (*F*(1, 27) = 0.02, *p* = 0.90, *η*_*p*_^2^ = 0.001; BF = 0.08) or delay (*F*(1, 27) = 0.002, *p* = 0.96, *η*_*p*_^2^ < 0.001; BF = 0.08) on YES responses. There were also no significant interaction effects between delay and ratio (*F*(3.03, 81.68) = 2.21, *p* = 0.09, *η*_*p*_^2^ = 0.08; BF < 0.01), between pain intensity and delay (*F*(1, 27) = 0.37, *p* = 0.55, *η*_*p*_^2^ = 0.014; BF < 0.01) or between pain intensity, delay and ratio (*F*(2.31, 62.35) = 0.70, *p* = 0.52, *η*_*p*_^2^ = 0.03; BF < 0.01) on YES responses. There was, however, a significant interaction effect between pain intensity and ratio (*F*(2.43, 65.57) = 3.05, *p* = 0.045, *η*_*p*_^2^ = 0.10).

To further investigate the interaction between pain intensity and ratio, the proportion of YES responses in the pain conditions was calculated averaging the YES responses in pain immediate and pain delayed testing conditions for each comparisons’ duration. Similarly, the proportion of YES responses in the no-pain conditions was calculated averaging the YES responses in no-pain immediate and no-pain delayed testing conditions (see Table 1). Visual inspection of Table 1 suggests that the proportion of YES responses for the shortest comparison (i.e., 0.625, 0.750 and 0.875) was higher in the no-pain conditions than in the pain conditions; meanwhile, the proportion of YES responses for the longest comparison (i.e., 1.125, 1.250 and 1.375) was lower in the no-pain conditions than in the pain conditions. This would suggest that pain related standards were perceived for longer than no-pain related standards. A paired-sample *t*-test between the no-pain and pain conditions was then conducted for each ratio (0.625, 0.750, 0.875, 1.000, 1.125, 1.250 and 1.375). To adjust for Type 1 error due to multiple comparisons, the number of comparisons (7) was taken into account: *p*-value was required to be < 0.0071 (= 0.05/7) to confirm significance. Paired-sample t-tests showed no significant difference between YES responses in the pain and no-pain conditions for the 0.625 (*p* = 0.18), 0.750 (*p* = 0.29), 0.875 (*p* = 0.019), 1 (*p* = 0.48), 1.125 (*p* = 0.31) and 1.250 (*p* = 0.17) ratio. The YES responses for the 1.375 ratio were, however, significantly higher in the pain conditions than in the no-pain conditions (*p* = 0.004) perhaps suggesting that the standard stimuli in the two pain conditions were perceived as subjectively longer than the standard stimuli in the two no-pain conditions.

**Table 1:** Proportion of YES responses (and standard deviation) averaged across the two no-pain conditions (no-pain immediate and no-pain delay) and across the two pain conditions (pain immediate and pain delay) in Experiment 1

Comparison/standard ratio	0.625	0.750	0.875	1	1.125	1.250	1.375
No-pain	0.20 (0.23)	0.39 (0.22)	0.65 (0.22)	0.67 (0.19)	0.57 (0.26)	0.39 (0.25)	0.19 (0.20)
Pain	0.13 (0.20)	0.32 (0.26)	0.21 (0.22)	0.69 (0.19)	0.62 (0.22)	0.45 (0.26)	0.31 (0.24)

#### Temporal accuracy

Table 2 shows temporal accuracy in the four conditions. Examination of Table 2 suggests that accuracy was similar in all conditions. A repeated measures ANOVA with pain intensity (no-pain vs pain) and delay (immediate vs delay) as within-subject factors confirmed these suggestions. There was no significant effect of pain intensity (*F*(1, 27) = 0.57, *p* = 0.46, *η*_*p*_^2^ = 0.02; BF = 0.24) nor delay (*F*(1, 27) = 1.75, *p* = 0.20, *η*_*p*_^2^ = 0.06; BF = 0.51) on accuracy. There was also no significant interaction effect between pain intensity and delay (*F*(1, 27) = 1.49, *p* = 0.23, *η*_*p*_^2^ = 0.05; BF = 0.06). Response accuracy was, therefore, unaffected by pain or delay.

**Table 2:** Means (and standard deviations) of accuracy, mid-three, peak time and FWHM in the four conditions (no-pain immediate, no-pain delay, pain immediate and pain delay) in Experiment 1

Condition	Accuracy	Mid-three	Peak time	FWHM
No-pain immediate	0.66 (0.11)	0.67 (0.14)	0.97 (0.15)	0.45 (0.14)
No-pain delay	0.61 (0.14)	0.56 (0.14)	0.97 (0.17)	0.46 (0.26)
Pain immediate	0.65 (0.12)	0.64 (0.17)	1.01 (0.16)	0.47 (0.17)
Pain delay	0.65 (0.11)	0.59 (0.16)	1.09 (0.23)	0.54 (0.30)

#### Response variability

Table 2 shows response variability (i.e., mid-three) in the four conditions. Examination of Table 2 suggests that variability was lower (i.e., gradients were more peaked) in the immediate testing than delayed testing conditions. A repeated measures ANOVA revealed a significant main effect of delay on mid-three (*F*(1, 27) = 8.41, *p* = 0.007, *η*_*p*_^2^ = 0.24). Mid-three was significantly higher in the immediate conditions compared to the delayed conditions suggesting that delay disrupts temporal discrimination of both pain and no-pain related stimuli. There was, however, no main effect of pain intensity (*F*(1, 27) = 0.001, *p* = 0.97, *η*_*p*_^2^ < 0.01; BF = 0.20) and no significant interaction effect between delay and pain intensity (*F*(1, 27) = 0.68, *p* = 0.42, *η*_*p*_^2^ = 0.03; BF = 0.82). Therefore, although delay per se increased response variability, pain itself did not affect response variability.

#### Peak time

Table 2 shows peak time in the four conditions. Examination of Table 2 suggests that peak time was slightly higher in the pain conditions than in the no-pain conditions. A repeated measures ANOVA with pain intensity (no-pain vs pain) and delay (immediate vs delay) as within-subject factors confirmed these suggestions. There was a significant effect of pain intensity on peak time (*F*(1, 27) = 10.73, *p* = 0.003, *η*_*p*_^2^ = 0.28); meanwhile, there was no effect of delay on peak time (*F*(1, 27) = 1.44, *p* = 0.24, *η*_*p*_^2^ = 0.05; BF = 0.36). There was also no significant interaction effect between pain intensity and delay (*F*(1, 27) = 1.08, *p* = 0.31, *η*_*p*_^2^ = 0.04; BF = 0.95). Response peak was, therefore, higher in the pain conditions than in the no-pain conditions, suggesting that pain had a lengthening effect on the perceived duration of the standard stimulus.

#### FWHM

Table 2 shows temporal FWHM in the four conditions. Examination of Table 2 suggests that FWHM was comparable across the four conditions. A repeated measures ANOVA with pain intensity (no-pain vs pain) and delay (immediate vs delay) as within-subject factors confirmed these suggestions. There was no significant effect of pain intensity (*F*(1, 27) = 1.27, *p* = 0.27, *η*_*p*_^2^ = 0.05; BF = 0.42) nor delay (*F*(1, 27) = 1.10, *p* = 0.30, *η*_*p*_^2^ = 0.04; BF = 0.33) on FWHM. There was also no significant interaction effect between pain intensity and delay (*F*(1, 27) = 0.84, *p* = 0.37, *η*_*p*_^2^ = 0.03; BF = 0.05). Response spread was, therefore, unaffected by pain or delay.

### Discussion

The results of Experiment 1 suggest that memory for the duration was largely unaffected when a low level of pain was experienced during the encoding of temporal information. This was confirmed by the absence of the effect of low pain on the measures of response accuracy, response variability and FWHM. The only effect of pain was seen when comparing responses to the longest of the comparison stimuli and on response peak. Critically, however, the pain effect was the same in the immediate and the delay condition, suggesting that memory for duration is unaffected by low pain during encoding. This contrasts with the expectations of the study: pain neither disrupted the cognitive resources necessary for correct memorization of duration, nor enhanced memory of events, as emotions do (Cocenas-Silva et al., 2012).

Memory for duration was, however, affected by delay, with significantly more variable responding in the delayed testing conditions than the immediate testing conditions (as indexed by the mid-three response variability measure). This replicates previous findings that memory for duration can decay over short delays (Lieving et al., 2006; Wearden & Ferrara, 1993; Wearden et al., 2002) and confirmed that the methodology was appropriate for detecting delay induced changes in responding.

One unexpected finding of the current study is that generalisation gradients were not systematically skewed by the presence of pain. Previous research shows that painful events are perceived as lasting for longer than neutral events (Piovesan et al., 2019). We may, therefore, have expected left skewed gradients (i.e., greater proportion of YES responses to durations longer than the standard). Although this was observed to some extent, that is the response peak was slightly higher in the pain conditions than in the no-pain conditions and the multiple comparisons showed that the 1.375 comparison was recognized more often as the standard in the painful than the neutral conditions, no differences were observed for other comparison durations or in measures of variability. One possibility is that pain did not have a clear and systematic effect on responding in this task, because the level of pain induced was not intense enough to affect responding. To test this possibility a further experiment was conducted using the same experimental design as Experiment 1 but with a greater pain intensity.

## Experiment 2

Experiment 2 used the same experimental design as Experiment 1; however, the level of pain induced was increased from low to high. Therefore, in the initial intensity rating task, participants were asked to select the thermal intensity that corresponded to high pain (6 in the NRS). The same possible outcomes anticipated in Experiment 1 were here expected.

### Method

#### Participants

Twenty-eight participants (21 females and 7 males; mean age = 24.11, SD = 4.83) were recruited. Participants were required not to be pregnant and not to have chronic pain, skin problems (e.g., eczema) or any impairment of body sensation. Additionally, they were asked not to take any analgesic during the 8 h prior to the experiment. Participants were reimbursed £5 in vouchers for taking part. The study was approved by the Liverpool John Moores University ethics committee and informed consent was obtained from all participants. The study was conducted in accordance with the declaration of Helsinki.

#### Procedure

Participants completed the same procedure used in Experiment 1. Only, participants selected thermal intensities equal to 0 and 6 in the NRS (instead of 0 and 3) during the intensity rating task, that is a warm but non painful intensity and a high pain intensity (following Khoshnejad et al., 2016). During the temporal generalisation task, therefore, participants felt the thermode being at high pain intensity during the training phase of the two pain conditions (pain immediate and pain delay). Experimental design and data analysis were as in Experiment 1.

### Results

Participants selected 36.88 °C (SD = 1.52) as warm intensity and 43.86 °C (SD = 1.54) as high pain intensity during the initial intensity rating task. A paired-sample t-test indicated that participants selected a significantly higher temperature in the pain condition than in the no pain condition (*t*(27) = 24.05, *p* < 0.001). An independent-sample t-test indicated that participants selected a significantly higher temperature for the pain condition in Experiment 2 than in Experiment 1 (*t*(54) = 2.77, *p* = 0.008).

**Fig. 2:**
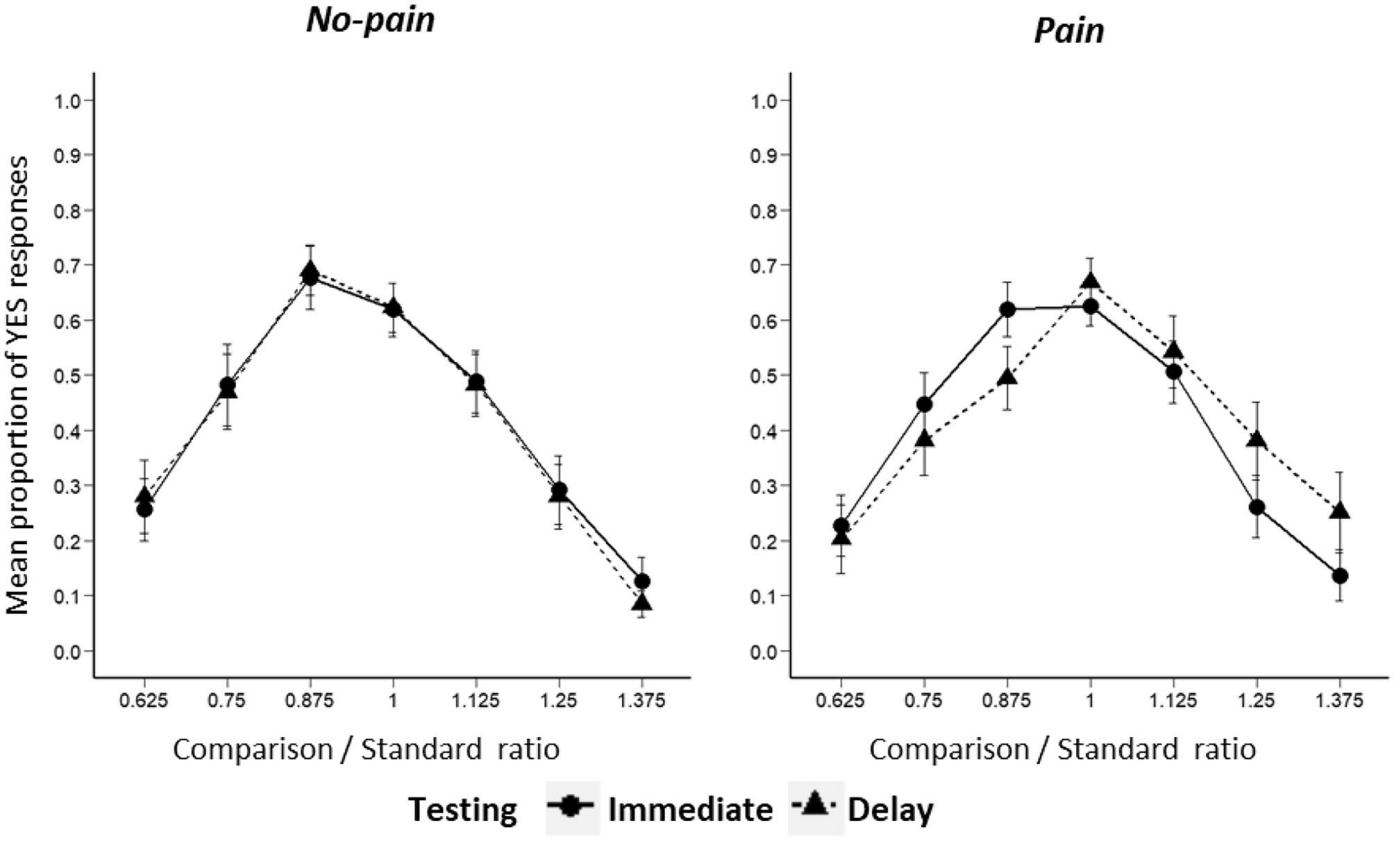
Proportion of YES responses plotted against comparison/standard ratio in Experiment 2. YES responses are divided between immediate (*solid line*) and delay (*dotted line*), and between no-pain (*left panel*) and pain (*right panel*)

Figure 2 shows temporal generalisation gradients depicting the mean proportion of YES responses in the four conditions plotted against comparison/standard ratio. Examination of Fig. 2 suggests that in the no pain condition, there was no effect of delay on responding. In the pain condition, gradients appear to be shifted to the right following the delay. A repeated measures ANOVA with pain intensity (no-pain vs pain), delay (immediate vs delay) and comparison/standard ratio (0.625, 0.750, 0.875, 1.000, 1.125, 1.250 or 1.375) as within-subject factors was conducted. The ANOVA showed significant main effects of ratio (*F*(2.21, 59.77) = 30.37, *p* < 0.001, *η*_*p*_^2^ = 0.53), but no main effect of pain intensity (*F*(1, 27) = 0.25, *p* = 0.62, *η*_*p*_^2^ = 0.01; BF = 0.08) nor delay (*F*(1, 27) = 0.12, *p* = 0.73, *η*_*p*_^2^ = 0.005; BF = 0.08) on the proportion of YES responses. There were also no significant interactions between delay and ratio (*F*(1.95, 52.60) = 0.72, *p* = 0.49, *η*_*p*_^2^ = 0.03; BF < 0.01), pain intensity and delay (*F*(1, 27) = 0.39, *p* = 0.54, *η*_*p*_^2^ = 0.01; BF < 0.01), pain intensity and ratio (*F*(1.79, 48.26) = 1.75, *p* = 0.19, *η*_*p*_^2^ = 0.06; BF < 0.01) nor between pain intensity, delay and ratio (*F*(1.88, 50.78) = 1.02, *p* = 0.37, *η*_*p*_^2^ = 0.04; BF < 0.01). YES responses were, therefore, unaffected by pain or delay.

#### Response accuracy

Table 3 shows response accuracy in the four conditions. Examination of Table 3 suggests that accuracy was similar in all conditions. A repeated measures ANOVA with pain intensity (no-pain vs pain) and delay (immediate vs delay) as within-subject factors confirmed these suggestions. There were no significant effects of pain intensity (*F*(1, 27) = 0.84, *p* = 0.37, *η*_*p*_^2^ = 0.03; BF = 0.35) nor delay (*F*(1, 27) = 0.52, *p* = 0.48, *η*_*p*_^2^ = 0.02; BF = 0.23) on accuracy. There was also no significant interaction effect between pain intensity and delay on accuracy (*F*(1, 27) = 0.10, *p* = 0.75, *η*_*p*_^2^ < 0.01; BF = 0.02). Response accuracy was, therefore, unaffected by pain or delay.

**Table 3:** Means (and standard deviations) of accuracy and mid-three in the four conditions (no-pain immediate, no-pain delay, pain immediate and pain delay) in Experiment 2

Condition	Accuracy	Mid-three	Peak time	FWHM
No-pain immediate	0.62 (0.13)	0.62 (0.16)	0.95 (0.15)	0.41 (0.013)
No-pain delay	0.62 (0.13)	0.63 (0.15)	0.93 (0.14)	0.43 (0.11)
Pain immediate	0.63 (0.11)	0.64 (0.16)	0.94 (0.16)	0.46 (0.16)
Pain delay	0.65 (0.13)	0.61 (0.18)	1.00 (0.18)	0.43 (0.14)

#### Response variability

Table 3 shows response variability (i.e., mid-three) in the four conditions. Examination of Table 3 suggests that variability was similar in all conditions. A repeated measures ANOVA with pain intensity (no-pain vs pain) and delay (immediate vs delay) as within-subject factors confirmed these suggestions. There were no significant effects of pain intensity (*F*(1, 27) = 0.04, *p* = 0.85, *η*_*p*_^2^ < 0.01; BF = 0.20) nor delay (*F*(1, 27) = 0.18, *p* = 0.68, *η*_*p*_^2^ < 0.01; BF = 0.21) on mid-three. There was also no significant interaction effect between pain intensity and delay on mid-three (*F*(1, 27) = 0.59, *p* = 0.45, *η*_*p*_^2^ = 0.02; BF = 0.01). Response variability was, therefore, unaffected by pain or delay.

#### Peak time

Table 3 shows peak time in the four conditions. Examination of Table 3 suggests that peak time was comparable across conditions. A repeated measures ANOVA with pain intensity (no-pain vs pain) and delay (immediate vs delay) as within-subject factors confirmed this suggestion. There was no significant effect of pain intensity (*F*(1, 27) = 0.93, *p* = 0.34, *η*_*p*_^2^ = 0.03; BF = 0.33) nor delay (*F*(1, 27) = 0.44, *p* = 0.51, *η*_*p*_^2^ = 0.02; BF = 0.23) on peak time. There was also no significant interaction effect between pain intensity and delay (*F*(1, 27) = 1.55, *p* = 0.22, *η*_*p*_^2^ = 0.05; BF = 0.05). The response peak was, therefore, unaffected by pain or delay.

#### FWHM

Table 3 shows temporal FWHM in the four conditions. Examination of Table 2 suggests that FWHM was similar in all conditions. A repeated measures ANOVA with pain intensity (no-pain vs pain) and delay (immediate vs delay) as within-subject factors confirmed these suggestions. There was no significant effect of pain intensity (*F*(1, 27) = 2.31, *p* = 0.14, *η*_*p*_^2^ = 0.08; BF = 0.46) nor delay (*F*(1, 27) = 0.08, *p* = 0.79, *η*_*p*_^2^ = 0.01; BF = 0.21) on FWHM. There was also no significant interaction effect between pain intensity and delay (*F*(1, 27) = 1.40, *p* = 0.25, *η*_*p*_^2^ = 0.05; BF = 0.05). The response peak was, therefore, not significantly affected by pain in our study.

### Discussion

Experiment 2 tested whether a higher level of pain intensity during the encoding of duration information would affect subsequent memory for duration. The proportion of YES responses, response accuracy, variability, peak and FWHM were similar between pain and no-pain conditions, suggesting that the high pain in our study had no significant effect on memory for duration. Unlike in Experiment 1, there was also no significant effects of delay on responding, contrasting previous studies that have found effects of shorter delay on perceived duration (Lieving et al., 2006; Wearden & Ferrara, 1993; Wearden et al., 2002).

## General discussion

The current study sought to establish the effect of low pain (Experiment 1) and high pain (Experiment 2) on the memorization of duration. Participants completed temporal generalisation tasks in which they encoded a standard duration while experiencing concurrent neutral or painful somatosensory stimulation. Participants then completed a recognition task in which they identified the standard duration from an array of comparison durations, in the absence of pain, either immediately after encoding or after a 15-min delay.

For both low and high pain intensities, when testing occurred immediately after the encoding of the standard duration, there were no consistent and systematic effects of pain on responses. There were no significant differences in response accuracy, response variability and FWHM between the immediate testing pain and no pain conditions. Although peak time was significantly higher in the low pain than no pain condition (Experiment 1), and there was a significantly greater proportion of YES responses to the longest comparison in the pain than no-pain condition (Experiment 1), there were no differences in responses for the other comparison durations. Furthermore, these findings were not replicated when a higher intensity pain stimulus was used (Experiment 2). This suggests that experiencing pain during stimulus encoding did not systematically affect the immediate recognition of temporal information. This supports Cocenas-Silva et al.'s (2012) findings, which showed that emotion did systematically not affect the temporal generalisation performance in a temporal generalisation task when the testing phase occurred immediately after the learning phase.

Comparison of the pain and no-pain delayed testing conditions showed a similar pattern of results. For participants exposed to low pain, peak time was higher in the pain delay than in the no-pain delay condition. However, there was no significant difference in the mean proportion of YES responses, response accuracy, response variability and response spread across the two conditions. For participants exposed to high pain, there was no significant difference in the mean proportion of YES responses, peak time, accuracy or response spread. Together these findings suggest that low and high pain do not have consistent and systematic effects on memory for duration. This contrasts with Cocenas-Silva et al. (2019) which showed that high stress levels were associated with more variable and less accurate temporal responses in a 24-h delayed temporal generalisation task. It also contrasts with Cocenas-Silva et al. (2012) which showed that memory for duration of emotional events is less vulnerable to distortion and decay than memory for duration of neutral events.

Collectively, the findings of Experiments 1 and 2 suggest that experiencing pain during the encoding of a temporal stimulus does not have a systematic effect on immediate or delayed recognition of this temporal stimulus. This finding contrasts with expectations that pain during encoding may impair future recognition due to distraction and disruption to cognition, or, enhance future recognition because of a pain-induced increase in the neurochemicals associated with consolidation in the hippocampus.

One possible explanation for the null effect of pain observed in the current study is that the delay imposed between learning and testing was not sufficient to reveal the effect of pain. While there is some evidence to suggest that memory consolidation can occur in a relatively short period of time, for example, within 12 min of encoding (Lechner et al., 1999), there is also evidence to suggest that consolidation takes significantly longer and is aided by sleep (see Walker et al., 2003; Stickgold, 2005 for discussion). Indeed, benefits of consolidation periods of longer than 1 h have been demonstrated specifically for duration (Cocenas-Silva et al., 2014) and previous studies demonstrating effects of emotion on memory for duration have sometimes imposed longer delays between learning and testing. For example, Cocenas-Silva et al. (2012, 2019) imposed a 24-h delay between learning and testing. It is, therefore, possible that a longer retention period would elicit an effect of pain on memory for duration. However, it should be noted that significant effects of short delays comparable to those used in this study have been reported in other studies. For example, a 10-s delay between learning and testing phases in temporal generalisation tasks has been found to impair temporal performance (e.g., Lieving et al., 2006; Wearden & Ferrara, 1993; Wearden et al., 2002). Future research should, however, consider the effect of longer delays when examining the effect of pain experience on duration stored in reference memory.

Another possibility is that the pain administered during this study did not affect memory for duration, because the pain was not itself task-relevant. Piovesan et al. (2019) demonstrated that task-relevancy determines pain distortions to perceived time during verbal estimation tasks; clear lengthening effects of pain on perceived duration were only observed when the to-be-timed stimulus was itself painful. Concurrent pain, which was not timed, did not distort perceived duration of a neutral to-be-timed stimulus. Similar observations have been made in studies examining the timing of non-painful emotional stimuli (Ogden et al., 2015).

Although task relevance offers an explanation as to why the standards encoded during pain were not remembered as systematically longer than the standards encoded during neutral somatosensory stimulation, it does not explain why pain did not disrupt the cognitive processes upon which memory consolidation are reliant. Pain akin to that administered in this study has been shown to impair attention (Moore et al., 2012; Van Damme et al., 2007), executive function (Moriarty et al., 2011), working memory (Dick & Rashiq, 2007) and recognition memory (Leavitt & Katz, 2006). Therefore, despite task irrelevant pain not disrupting the timing process itself, there is good evidence to suggest that it should have disrupted the memorization process, leading to poorer recognition performance.

This raises the possibility that duration is somehow “protected” from the disruptive effects of pain on memory. At present, it is unclear why this would be. One possibility is that in the same way in which emotional distortions to time are thought to have an adaptive origin (see Droit-Volet & Gil, 2009; Lake, LaBar, & Meck, 2016; Piovesan et al., 2019 for discussion), there may be an adaptive origin to duration information encoded during pain or injury being protected from disruption. Indeed, although pain has a substantial affective component, pain is often not classified as an emotion per se, but is considered a complex biopsychosocial construct, where physiological, social, affective and cognitive dimensions all interact to create the experience of pain (Engel, 1980). The multifaceted nature of pain experience may, therefore, contribute towards it preventing disruption to distortion to temporal information. Future research should, therefore, examine more broadly the effects of pain on memory for duration.

A final possibility is that the length of the durations used minimised the effect of pain on their memorization. In the current study, the standard durations were all less than 1 s long. Short, sub-second durations are thought to be processed by different neural circuits to longer multiple second intervals (see Lewis & Miall, 2003a, 2003b for discussion). Most notably, while the timing of short epochs is thought to place relatively little demand on sustained attentional processing, the timing of longer multi-second or multi-minute epochs requires significantly greater sustained attentional processing (Lewis & Miall, 2003a, 2003b). Pain experience disrupts sustained attentional processing (see Eccleston & Crombez, 1999) and it is, therefore, possible that pain may have a greater effect on the memorization of longer durations due to the effect of pain on the sustained attentional resources required to process these epochs. Future research should, therefore, explore the effect of pain on longer duration ranges.

## Limitations

Pain experience differs between individuals and in particular between genders. For example, pain threshold, tolerance and sensitivity differ across sexes (Bartley & Fillingim, 2013; Berkley, 1997; Wiesenfeld-Hallin, 2005). It is, therefore, possible that gender differences in pain experience may influence the effect of pain on memory for duration. Although the current study used an intensity rating task to ensure subjectively similar pain experience across participants, it remains possible that gender differences in pain may have influenced the effect of pain on memory for duration. Due to the small number of male participants in the current study, it was not possible to test this conclusively. Future research should, therefore, seek to establish whether there are gender differences in the effect of pain on immediate and delayed temporal processing.

It was not possible to establish whether there were systematic order effects in the current study which may have affected learning, memory interference or vigilance across the conditions. However, the likelihood of order effects was reduced, because the duration of the standard was selected at random from a distribution (400–800 ms) for each participant in each condition. Furthermore, the order of the conditions was counterbalanced across participants resulting in 24 potential condition orders.

## Conclusions

While previous studies examined the effect of pain on perceived duration testing participants’ perception immediately after the pain presentation (see Fayolle et al., 2015; Ogden, Moore, et al., 2014; Ogden, Wearden, et al., 2014; Piovesan et al., 2019; Rey et al., 2017), this study was the first to examine the effect of pain on perceived duration when participants are required to store the duration in reference memory over periods of delay. The results suggest that, unlike high stress levels, which decreases temporal performances after a delay, pain does not appear to disrupt memory for duration. This raises the possibility that duration processing is somehow protected from the impairing effects of pain on memory and cognition noted in non-temporal fields.

## Data Availability

Datasets of Experiments 1 and 2 have been made publicly available at https://osf.io/4vazn/?view_only=8031bef6a4b24fa89229389f86781229.
